# Constraints to sustainable energy technology implementation: Insights from an emerging economy using ISM-MICMAC analysis

**DOI:** 10.1371/journal.pone.0331334

**Published:** 2025-09-03

**Authors:** Zhengguang Shi, Bingyao Li, Raluca Simina Bilti

**Affiliations:** 1 College of Business Administration College, Chaohu University, Hefei, China; 2 College of Business, University Utara Malaysia, Sintok Kedah Darul Aman, Malaysia; 3 School of Economics and Management, China University of Mining and Technology, Xuzhou, China; 4 Jiangsu Union Technical Institute, Xuzhou Vocational Technology Academy of Finance and Economics, Xuzhou, China; 5 Faculty of Economics, Aurel Vlaicu University of Arad, Arad, Romania; Kwara State University, NIGERIA

## Abstract

The growing environmental, economic, and social challenges have compelled governments and policymakers to incorporate sustainable energy technologies. However, the development of sustainable energy technologies is hindered by various barriers in emerging economies like China. Although, previous studies attempted to investigate these barriers but there is dearth of studies determining the hierarchy ranking of the barriers. ISM based approach has been developed to fill this research gap by presenting the barriers on the basis of importance level. Through literature survey, fourteen barriers have been identified which were confirmed from expert’s panel. These fourteen barriers are displayed in six layers of ISM hierarchy form from bottom to top which exhibits their ranking on priority base. Findings revealed that high initial cost, longer payback period, and lack of proper financing facilities are major barriers in adopting sustainable energy technologies. However, lack of social acceptance is the least important barrier in sustainable energy technologies. This work will assist companies to better understand the relationships between the barriers which is useful to reduce the barriers strength by implementing effective strategies.

## Introduction

The global warming needs urgent and unprecedented action and one of the most effective solutions of climate change is the widespread adoption of sustainable energy technologies [1]. The sustainable technologies are divided into two categories: adaptation technologies or mitigation technologies. The goal of mitigation technologies, such as solar and wind energy and biofuels is to mitigate the negative repercussions of climate change by minimizing greenhouse gas and carbon emissions, coal use, and carbon output. However, adaptation technologies include space reflectors and stratospheric aerosol treatments concentrating on changing human behavior and the environment to adapt to climate change. These adaptive technologies are classified as either geoengineering or environmental engineering [2]. These eco-friendly technologies are parts of a circular economy or sustainable energy sources which aims to reduce greenhouse gas emissions that could significantly enhance the quality of life [3]. Also, several studies have found that sustainable energy technologies have a significant positive impact on social prosperity and economic development [4].

Since sustainable energy are easily accessible and produced from natural resources so, many researchers recommended to incorporate these technologies to save money and protect the environment. However, integrating sustainable energy technologies (SETs) into a nation’s energy strategy is a complicated and challenging task [5]. Some authors also contend that a sociotechnical transformation requires a change in the existing rules and technological frameworks therefore, this transition is expensive and time-consuming. To overcome this issue, the implementation of sustainable energy technologies requires appropriate policies and social awareness campaigns [6]. Some contextual factors are hindering the development of sustainable technologies. Therefore, the green energy technologies adoption can be influenced based on each country technical ability, socioeconomic situation, geographical position, and its political environment. The formulation and implementation of sustainable energy policies are often delayed as a result of the lack of contextual based studies. A clear understanding of the nature of the barriers is essential to overcome them [7,8]. Densely populated developing countries with limited access to clean energy can greatly benefit from renewable energy sources [9]. The need for economical, sustainable, and environmental friendly energy systems in emerging economies is more apparent due to high population density and large energy consumption [10]. Latest research indicates that conventional energy usage in rural areas significantly contributes to greenhouse gas emissions [11].

China’s dependence on coal has made it the world’s largest carbon emitter, contributing to over 30% of global carbon emissions [12]. Recognizing the importance of sustainable energy, the Chinese government has set ambitious goals to reduce carbon emissions and increase the share of green energy in the energy portfolio [13]. China’s transition to sustainable energy faces several obstacles and among them major one is technological constraints [14]. Sustainable energy technologies need continuous research and development to improve efficiency and reduce costs. Another barrier impeding China’s green energy innovation is the lack of finance. Acquiring initial capital for sustainable energy projects is a challenging task for small and medium-sized firms [15]. Lack of funding and research and development for sustainable energy technology can also slow down the development of new innovations. Policy limitations are another obstacle to China’s green energy system. Although, previous work has contributed to sustainable energy technologies but factors hindering their adoption has narrowly researched [16]. Also, there are lack of studies which has established hierarchy relationships among barriers of sustainable energy technology.

This work contributes to the body of literature in three distinct ways. 1. This study provides a key foundation for the government, legislators, managers, and private sector which are engaged in planning and creating inclusive sustainable energy projects in developing nations such as China. 2. Identifying and ranking the most critical barriers to the development of sustainable energy in China is the paper’s most fundamental contribution. 3. A thorough literature review on the barriers to the development of sustainable energy has been presented in this study which can help the interested researchers in this field to overcome the obstacles.

## Literature review

One of the most pressing challenges being faced by industrialized and emerging nations is efficient energy utilization. Countries must create and deliver energy to achieve political, economic, and technical objectives [17]. The availability of green energy sources in rural and distant regions at affordable cost is big concern [18,19]. A well-designed technology that meets high energy demands without negatively impacting the planet is considered sustainable energy [20]. There are different kinds of sustainable and renewable energy technologies available in the market [21] such as, solar, thermal, low-energy cooling methods, geothermal, wind, photovoltaic cells, and bioenergy. The another terminology ‘renewable energy’ is considered a clean and affordable technology [22]. A subcategory of sustainable energy, renewable energy technologies—like solar, wind, and hydro—rely on resources that naturally replenish themselves. While all renewable energy is typically sustainable but all sustainable energy is not necessarily renewable. For instance, improved energy efficiency or the use of low-carbon technology are sustainable but non-renewable energy. The adoption of sustainable and renewable energy technologies is hindered by a number of obstacles, particularly related to regional and technological factors. Sustainable and renewable energy technologies are used simultaneously therefore, most of the barriers exhibit similarities in nature so, we consider all those factors which influence sustainable and renewable energy technologies adoption. Sustainable energy technologies (SETs) require significant investments, infrastructure and regulatory support [23].

Some studies claim that firms may not embrace sustainable energy technologies due to relatively long payback period and unpredictable market situations [24,25]. Using AHP approach, the author found that the successful execution of sustainable energy investments is hindered due to regulatory, political, social, financial and market-related barriers. Another author also found that low economic support, strict rules and regulations and volatile political system negatively impact sustainable energy technologies development in the region. The study based on Ghana identified 19 barriers of sustainable energy technologies adoption which is classified into six categories: market-related, economic and financial, technical, legal & regulatory framework, human skills, and sociocultural constraints. The findings suggest that low financial, economic, human, and technical skills were most prominent barriers [26]. With the help of Analytical Network Process (ANP), the study investigated that high investment and operating costs, poor public and private sectors partnership, and inadequate planning are the most critical barriers to the adoption of sustainable energy sources.

The slow commercialization of sustainable energy technologies in Finland is hindered by limited financing options, market uncertainty, infrastructural support, internationalization, and market-driven technology development [24,27]. Most of the scholarly work focuses on market related obstacles to the adoption of sustainable energy technologies [28]. High initial prices [29], high energy costs [30,31], R&D spillover [32], and small incentives [33] are typical barriers to the adoption of sustainable energy technologies. Few market-related barriers can also significantly restrict the expansion of sustainable energy technologies [34]. Most of the scholarly work [35–37] described several types of institutional barriers that prevents the adoption of sustainable energy technologies but market related barriers were neglected which should be explored [14]. Some of prominent market related barriers are lack of finance, shortage of time and resources and clear communication to take prompt decisions regarding the sustainable energy technologies acquisitions [38,39]. Based on prior literature, the sustainable energy technologies adoption depends on political, geographic, technological, socioeconomic and technical factors [8]. The sustainable energy technologies would not be implemented effectively without recognizing their challenges. Therefore, identifying and understanding the barriers relationships that influence the sustainable energy technologies adoption is important which needs to be addressed. The presentation of barriers in ranking form can assist managers in implementing sustainable energy technologies through designing suitable strategies. While barriers to sustainable energy technologies has been widely studied, but studies focused on Chinese context is rare [40].

Therefore, current study attempts to investigate the barriers associated with the adoption of sustainable energy technology in the Chinese manufacturing sector. The following are the specific objectives of the current study:

- To explore the barriers inhibiting the Chinese manufacturing sector from using sustainable energy technologies.
- To analyze the contextual relationship between identified barriers using ISM and MICMAC.
- To investigate barriers of sustainable energy technologies on the level of importance.

## Research methodology

To explore the barriers to sustainable energy technologies in manufacturing sector, interpretive structural modelling is employed. The ability of this approach is to transform poor articulated models and vague ideas into clear, understandable and well-defined model. ISM is particularly helpful to identify a group of variables that are either directly or indirectly connected in order to create a hierarchical structure. ISM illustrates the links between factors and provide more insightful information in a complicated decision-making setting [41,42].

The current study took manufacturing industry as a case sector. Four manufacturing companies were selected such as leather, pharmaceutical, textile and cement which are mainly responsible for massive environmental degradation and energy consumptions [43,44]. The literature screening regarding barriers of sustainable energy technologies adoption was performed from different databases in September 2023. Distinctive scholarly databases, including “Emerald Insight, Science Direct, Springer, Wiley Online, and Taylor and Francis,” were searched and initially identified 14 barriers. The main terminologies utilized for the article investigated were “green technology barriers,” “sustainable energy technologies barriers,” OR “sustainable energy technologies challenges,” OR “sustainable energy technologies handicaps,” OR “sustainable energy technologies issues,” OR “sustainable energy technologies obstacles,” “sustainable energy technologies barriers in developing countries,” and “sustainable energy technologies barriers in China.” The titles and abstracts of the listed articles were used to shortlist papers using the selected keywords after additional articles were eliminated. The most relevant articles from the shortlisted list were retained for full-text reading and referencing, and an improved list of sustainable energy technologies barriers was created which is shown in Table 1.

**Table 1:** An overview of barriers hampering sustainable energy technologies adoption.

Serial No.	Barriers	Description	References
(B1)	High initial capital	High initial capital costs can deter manufacturing firms from adopting sustainable energy technologies because the upfront investment required for infrastructure, equipment, and installation is often substantial, straining financial resources.	[45–50]
(B2)	Longer payback period	Firms in developing countries often short-term benefits instead of long-term benefits. As sustainable energy technologies provide long term benefits so, many firms hesitate to invest in such technologies.	[51–54]
(B3)	Ecological conditions	Ecological conditions, such as limited sunlight, low wind speeds, or unsuitable land, can make certain sustainable energy technologies like solar panels or wind turbines less effective or economically unviable in specific regions. Firms operating in these areas may avoid adoption due to low energy yields and poor return on investment.	[8,55]
(B4)	Lack of proper financing	Sustainable energy technologies adoption requires heavy investment. Hence, limited financing can restrain the ability of firms to acquire latest technologies and equipment’s.	[25,56–61]
(B5)	Lack of clear communication	Businesses may not completely comprehend the operational, financial, or environmental advantages of sustainable energy technologies if there is unclear communication, which can prevent them from implementing them.	[51,62,63]
(B6)	Lack of skilled personnel	Companies may be less inclined to invest in new technologies if they lack the technical know-how, since they may be more vulnerable to system failures or inefficiencies.	[57,64,65]
(B7)	Insufficient knowledge	Lack of information may discourage businesses from implementing sustainable energy technology because they might not be aware of the possible cost reductions, or the environmental advantages.	[8,46,47,66–71]
(B8)	Lack of social acceptance	Companies may be deterred from implementing sustainable energy technology by a lack of social acceptance because they fear criticism from the public.	[72–74]
B0	Policy and regulatory issues	Businesses may find it difficult to implement sustainable energy solutions when policy and regulations are unclear, inconsistent, or lack supportive frameworks like tax incentives, subsidies, or clear rules,	[64,75–80]
B10	Lack of energy storage technologies	Lack of efficient energy storage technologies limits firms’ ability to store excess energy generated from renewable sources like solar or wind, leading to reliability issues during periods of low generation.	[81–84]
B11	Lack of research facilities	Insufficient research facilities hinder businesses’ capacity to create, evaluate, and modify sustainable energy technology to suit their unique requirements or regional circumstances.	[53,60,77,78,85]
B12	Lack of consumer paying capacity	Lack of consumer paying capacity can discourage firms from adopting sustainable energy technologies because they may fear insufficient demand or an inability to pass on higher initial costs to price-sensitive consumers.	[45,46,78,86]
B13	Technology complexity	Since sustainable energy solutions frequently need for specific knowledge, skills, and training that may not be easily accessible inside the company due to complex nature which might deter businesses from using them.	[58,87]
B14	Lack of trust and reliability on energy sources	Concerns about irregular energy supplies and possible operational interruptions might deter businesses from adopting sustainable energy sources and technology if there is a lack of confidence in their dependability.	[85,88,89]

There are few steps of ISM techniques as follows

**Fig 1:**
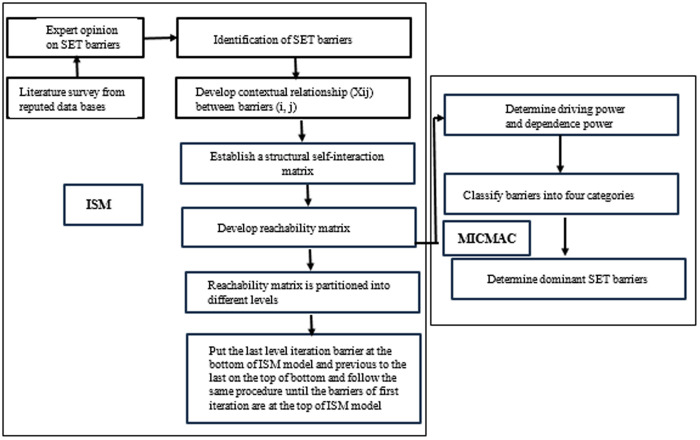
Flow diagram of ISM-MICMAC approach.

Step 1: This initial phase selects the barriers of sustainable energy technologies and then confirms from subject-matter experts.
Step 2: Establish contextual relationship among identified barriers of sustainable energy technologies.
Step 3: A structural self-interaction matrix (SSIM) is developed between two sustainable energy technologies barriers, i and j.
Step 4: SSIM is followed by IRM (initial reachability matrix), which substitutes binary digits 0 and 1in SSIM. ISM’s popular transitivity rule states if A is connected to B and B to C, then A is definitely related to C.
Step 5: The dependence and driving power of barriers can be obtained from the FRM (final reachability matrix) after applying rule of transitivity.
Step 6: Several partitions are derived with the use of FRM by comparing reachability and intersection set.
Step 7: Transitivity linkages are removed while drafting the FRM digraph (directed graph) relationship.
Step 8: Once nodes have been converted to digraphs and replaced with statements, ISM’s hierarchical model is developed.
Step 9: The theoretical interpretative structural model is retested in the case of difference to ensure reliable results. The ISM methodological framework has been shown in Fig 1.

### Structural self-interaction matrix (SSIM)

A review of the literature and the opinions of experts have been used to establish contextual relationship between the barriers to sustainable energy technologies. The expert panel conducted pairwise comparisons to determine the contextual relationship.

The SSIM was developed using the following four symbols (Table 2).

**Table 2:** SSIM.

Constructs	B1	B2	B3	B4	B5	B6	B7	B8	B9	B10	B11	B12	B13	B14
High initial capital (B1)	X	O	O	O	O	V	O	V	V	O	V	V	O	O
Longer payback period (B2)		X	O	O	O	V	V	V	V	O	V	O	V	O
Ecological conditions (B3)			X	A	O	V	V	V	X	V	V	V	O	O
Lack of proper financing (B4)				X	O	V	V	V	O	O	O	V	V	O
Lack of clear communication (B5)					X	O	V	V	O	O	V	V	O	O
Lack of skilled personnel (B6)						X	X	V	A	X	V	O	V	V
Insufficient knowledge (B7)							X	V	A	A	A	A	V	V
Lack of social acceptance (B8)								X	A	A	A	A	A	A
Policy and regulatory issues (B9)									X	O	V	V	V	O
Lack of energy storage technologies	(B10)									X	V	X	X	X
Lack of research facilities (B11)											X	A	A	O
Lack of consumer paying capacity (B12)												X	X	O
Technology complexity (B13)													X	O
Lack of trust and reliability on energy sources (B14)														X

**V**—When variable i help achieve variable j,
**O**—When variable i and j are unrelated,
**X**—When variable i and j help achieve one another,
**A**—When variable j helps achieve variable i.

For getting responses, a cover letter explaining the purpose of the research was shared. As the ISM methodology requires a minimum of eight experts, so the sample size used in this study is methodologically adequate. A study questionnaire (see SI) was created and sent to a panel of experts to elucidate the linkages among barriers. The experts chosen for this study were highly experienced and professional. Each of the expert possessed more than ten years of working experience in concerned field. The profile of expert’s members is presented in Table 3.

**Table 3:** Biodata of the expert’s team.

S. No	Position	Gender	Experience (Years)	Education
1	Community energy consultant	Male	15	PhD
2	Manufacturing specialist	Male	12	Master
3	Environmental policy officer	Female	11	PhD
4 5	Safety advisor Government official	Male Female	13 14	Bachelor PhD
6 7	Energy policy maker Consumer	Male Male	13 12	Bachelor Bachelor
8	Academician	Female	13	PhD
9	Academician	Male	14	PhD

### Initial reachability matrix (IRM)

The following guidelines explains how SSIM is changed to initial reachability matrix (IRM).

- If two initiatives (i and j) represent the symbol V, the conversion rule suggests setting the values of i and j to 1 and j and i to 0.
- If two initiatives (i and j) represent the letter A, the conversion rule suggests setting the values of i and j to 0 and j and i to 1.
- If two initiatives (i and j) represent the symbol X, then the conversion rule suggests setting the values of i and j to 1 and j and i to 1.
- If two initiatives (i and j) represent the symbol O, the conversion rule suggests setting the values of i and j to 0 and j and i to 0. The outcome of this process is shown in Table 4.

**Table 4:** Initial reachability matrix.

Constructs	B1	B2	B3	B4	B5	B6	B7	B8	B9	B10	B11	B12	B13	B14
High initial capital	1	0	0	0	0	1	0	1	1	0	1	1	0	0
Longer payback period	0	1	0	0	0	1	1	1	1	0	1	0	1	0
Ecological conditions	0	0	1	0	0	1	1	1	1	1	1	1	0	0
Lack of proper financing	0	0	1	1	0	1	1	1	0	0	0	1	1	0
Lack of clear communication	0	0	0	0	1	0	1	1	0	0	1	1	0	0
Lack of skilled personnel	0	0	0	0	0	1	1	1	0	1	1	0	1	1
Insufficient knowledge	0	0	0	0	0	1	1	1	0	0	0	0	1	1
Lack of social acceptance	0	0	0	0	0	0	0	1	0	0	0	0	0	0
Policy and regulatory issues	0	0	1	0	0	1	1	1	1	0	1	1	1	0
Lack of energy storage technologies	0	0	0	0	0	1	1	1	0	1	1	1	1	1
Lack of research facilities	0	0	0	0	0	0	1	1	0	0	1	0	0	0
Lack of consumer paying capacity	0	0	0	0	0	0	1	1	0	1	1	1	1	0
Technology complexity	0	0	0	0	0	0	0	1	0	1	1	1	1	0
Lack of trust and reliability on energy sources	0	0	0	0	0	0	0	1	0	1	0	0	0	1

### Final reachability matrix (FRM)

In this step, the transitivity rule is applied to transform IRM to the final reachability matrix (FRM). According to the transitivity rule, if A influences B and B influences C, then C should influence A. The final reachability matrix created by including the transitivity idea

which is displayed in Table 5. In the end, the final reachability matrix is used to calculate driving and dependency power.

**Table 5:** FRM.

Constructs	B1	B2	B3	B4	B5	B6	B7	B8	B9	B10	B11	B12	B13	B14	Driving power
B1	1	0	1*	0	0	1	1*	1	1	1*	1	1	1*	1*	11
B2	0	1	1*	0	0	1	1	1	1	1*	1	1*	1	1*	11
B3	0	0	1	0	0	1	1	1	1	1	1	1	1*	1*	10
B4	0	0	1	1	0	1	1	1	1*	1*	1*	1	1	1*	11
B5	0	0	0	0	1	1*	1	1	0	1*	1	1	1*	1*	9
B6	0	0	0	0	0	1	1	1	0	1	1	1*	1	1	8
B7	0	0	0	0	0	1	1	1	0	1*	1*	1*	1	1	8
B8	0	0	0	0	0	0	0	1	0	0	0	0	0	0	1
B9	0	0	1	0	0	1	1	1	1	1*	1	1	1	1*	10
B10	0	0	0	0	0	1	1	1	0	1	1	1	1	1	8
B11	0	0	0	0	0	1*	1	1	0	0	1	0	1*	1*	6
B12	0	0	0	0	0	1*	1	1	0	1	1	1	1	1*	8
B13	0	0	0	0	0	1*	1*	1	0	1	1	1	1	1*	8
B14	0	0	0	0	0	1*	1*	1	0	1	1*	1*	1*	1	8
Dependence power	1	1	5	1	1	13	13	14	5	12	13	12	13	13	117

### Level partitioning

Level partitioning is used to get the prominence of each barrier. The reachability set and antecedent set are now obtained using the reachability matrix. The reachability set includes both the variable itself and the group of variables that are impacted by it. In the antecedent set, the collection of factors that impact it and the variable itself are taken. All of the variables’ different sets including reachability, antecedent, and intersection sets are acquired. The barrier that first achieves the same reachability and intersection set is given level 1 in the ISM model. The same process is repeated again and this iterative procedure is continued until all barriers have not attained levels (see Tables 6–11). Later, transitivity is eliminated and a hierarchy-based digraph structure is created. The discussion section provides further information on the ISM model.

**Table 6:** Partitioning levels of sustainable energy technologies barriers.

Constructs	Reachability set	Antecedent set	Intersection set	Level
	R (Ci)	A (Cj)	I = R (Ci) ∩ A (Cj)	
B1	B1, B3, B6, B7, B8, B9, B10, B11, B12, B13, B14	B1	B1	
B2	B2, B3, B6, B7, B8, B9, B10, B11, B12, B13, B14	B2	B1	
B3	B3, B6, B7, B8, B9, B10, B11, B12, B13, B14	B1, B2, B3, B4, B9	B3, B9	
B4	B3, B4, B6, B7, B8, B9, B10, B11, B12, B13, B14	B4	B4	
B5	B5, B6, B7, B8, B10, B11, B12, B13, B14	B5	B5	
B6	B6, B7, B8, B10, B11, B12, B13, B14	B1, B3, B6, B7, B9, B10, B11, B12, B13, B14	B6, B7, B10, B11, B12, B13, B14	
B7	B6, B7, B8, B10, B11, B12, B13, B14	B1, B3, B6, B7, B9, B10, B11, B12, B13, B14	B6, B7, B10, B11, B12, B13, B14	
B8	B8	B1, B3, B6, B7, B8, B9, B10, B11, B12, B13, B14	B8	I
B9	B3, B6, B7, B8, B9, B10, B11, B12, B13, B14	B1, B2, B3, B4, B9	B9	
B10	B6, B7, B8, B9, B10, B11, B12, B13, B14	B1, B2, B3, B4, B5, B6, B7, B9, B10, B12, B13, B14	B6, B7, B10, B12, B13, B14	
B11	B6, B7, B8, B11, B13, B14	B1, B2, B3, B4, B5, B6, B7, B9, B10, B11, B12, B13, B14	B6, B7, B11, B13, B14	
B12	B6, B7, B8, B10, B11, B12, B13, B14	B1, B2, B3, B4, B5, B6, B7, B9, B10, B12, B13, B14	B6, B7, B10, B12, B13, B14	
B13	B6, B7, B8, B10, B11, B12, B13, B14	B1, B2, B3, B4, B5, B6, B7, B9, B10, B11, B12, B13, B14	B6, B7, B10, B11, B12, B13, B14	
B14	B6, B7, B8, B10, B11, B12, B13, B14	B1, B2, B3, B4, B5, B6, B7, B9, B10, B11, B12, B13, B14	B6, B7, B10, B11, B12, B13, B14	

**Table 7:** Partitioning.

Constructs	Reachability set	Antecedent set	Intersection set	Level
	R (Ci)	A (Cj)	I = R (Ci) ∩ A (Cj)	
B1	B1, B3, B6, B7, B9, B10, B11, B12, B13, B14	B1	B	
B2	B2, B3, B6, B7, B9, B10, B11, B12, B13, B14	B2	B2	
B3	B3, B6, B7, B9, B10, B11, B12, B13, B14	B1, B2, B3, B4, B9	B3, B9	
B4	B3, B4, B6, B7, B9, B10, B11, B12, B13, B14	B4	B4	
B5	B5, B6, B10, B11, B12, B13, B14	B5	B5	
B6	B6, B7, B10, B11, B12, B13, B14	B1, B2, B3, B4, B5, B6, B7, B9, B10, B11, B12, B13, B14	B6, B7, B10, B11, B12, B13, B14	II
B7	B6, B7, B10, B11, B12, B13, B14	B1, B2, B3, B4, B5, B6, B7, B9, B10, B11, B12, B13, B14	B6, B7, B10, B11, B12, B13, B14	II
B9	B3, B6, B7, B9, B10, B11, B12, B13, B14	B1, B2, B3, B4, B9	B9	
B10	B3, B6, B10, B11, B12, B13, B14	B1, B2, B3, B4, B9, FK, ATM, FS, M, FTO, SE, SC	B6, B7, B10, B12, B13, B14	
B11	B3, B6, B11, B13, B14	B1, B2, B3, B4, B5, B6, B7, B9, B10, B11, B12, B13, B14	B6, B7, B11, B13, B14	II
B12	B6, B7, B10, B11, B12, B13, B14	B1, B2, B3, B4, B5, B6, B7, B9, B10, B12, B13, B14	B6, B7, B10, B12, B13, B14	
B13	B6, B7, B10, B11, B12, B13, B14	B1, B2, B3, B4, B5, B6, B7, B9, B10, B11, B12, B13, B14	B6, B7, B10, B11, B12, B13, B14	II
B14	B6, B7, B10, B11, B12, B13, B14	B1, B2, B3, B4, B5, B6, B7, B9, B10, B11, B12, B13, B14	B6, B7, B10, B11, B12, B13, B14	II

**Table 8:** Partitioning.

Constructs	Reachability set	Antecedent set	Intersection set	Level
	R (Ci)	A (Cj)	I = R (Ci) ∩ A (Cj)	
B1	B1, B3, B9	B1	B1	
B2	B2, B3, B9	B2	B2	
B3	B3, B9	B1, B2, B3, B4, B9	B3, B9	IV
B4	B3, B4, B9	B4	B4	
B5	B5	B5	B5	IV
B9	B3, B9	B1, B2, B3, B4, B9	B9	

**Table 9:** Partitioning.

Constructs	Reachability set	Antecedent set	Intersection set	Level
	R (Ci)	A (Cj)	I = R (Ci) ∩ A (Cj)	
B1	B1, B2, B9, B10, B12	B1	B1	
B2	B2, B2, B9, B10, B12	B2	B2	
B3	B3, FS, M, FTO	B1, B2, B3, B4, B9	IB3, B9	
B4	B3, B4, B9, B10, B12	B4	B4	
B5	B5, B10, B12	B5	B5	
B9	B3, B9, B10, B12	B1, B2, B3, B4, B9	B9	
B10	B10, B12	B1, B2, B3, B4, B5, B9, B10, B12	B10, B12	III
B12	B10, B12	B1, B2, B3, B4, B5, B9, B10, B12	B10, B12	III

**Table 10:** Partitioning.

Constructs	Reachability set	Antecedent set	Intersection set	Level
	R (Ci)	A (Cj)		
			I = R	
			(Ci) ∩ A	
			(Cj)	
B1	B1, B9	B1	B1	
B2	B2, B9	B2	B2	
B4	B4, B9	B4	B4	
B9	B9	B1, B2, B4, B9	B9	V

**Table 11:** Partitioning.

Constructs	Reachability set	Antecedent set	Intersection set	Level
	R (Ci)	A (Cj)	I = R (Ci) ∩ A	
			(Cj)	
B1	B1	B1	B1	VI
B2	B2	B2	B2	VI
B4	B4	B4	B4	VI

### MICMAC analysis

**Fig 2:**
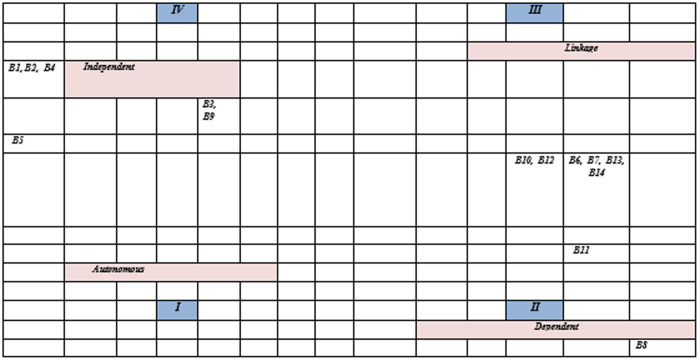
MICMAC analysis.

The cross-impact matrix multiplication commonly referred as MICMAC is applied for the purpose of classification [90]. Based on the driving and dependence power, MICMAC analysis is divided into four categories as shown in Fig 2: linkage, autonomous, independent, and dependent barriers.

#### Autonomous barriers.

This cluster contains barriers that have weak dependence and weak driving power. This study does not have any barriers in the autonomous cluster.

#### Linkage barriers.

This group of barriers has a strong driving and dependency power. There are six barriers in linkage cluster such as B6, B7, B13, B14, B10, B12.

#### Dependent barriers.

This cluster consists of barriers that have weak driving power but have strong dependence power. There are only two barriers like, B8 and B11 in the dependent cluster.

#### Independent barriers.

Barriers in this cluster are driven by strong driving but weak dependence power. In this cluster, there are six barriers; B1, B2, B3, B4, B5 and B9.

### Study results

The findings of the study declare that high initial capital, longer payback period, and lack of proper financing facilities are the most prominent barriers that discourage the adoption of sustainable energy technologies in the manufacturing sector [91,92]. Because of their high driving power, these three barriers are located at level 6 in the ISM hierarchy structure. Some companies have no access to incentives or accessible funds to acquire the initial capital.

Government subsidies, rebates on taxes, and low-interest loans are available in some areas but they might not always be adequate or well designed to meet the whole capital needs of sustainable energy projects. A large initial capital is required for many sustainable energy technologies (e.g., installing solar panels, improving HVAC systems, adopting energy storage solutions) [93]. Even green technology offers long-term benefits but businesses might not be able to incorporate without sufficient funding [94]. A longer payback period is also a significant barrier to implement green energy technologies in businesses since companies prioritize investments that yield quick returns [95]. If the cost of implementing sustainable energy solutions takes years to recover, companies may be reluctant to make the investment [86,96].

Next, at level fifth, the study found that policy and regulatory barriers impede manufacturing firms to adopt sustainable energy technologies. Previous studies also documented that lack of good governance is a fundamental barrier in the implementation phase of sustainable energy technologies [75,76]. Firms are often reluctant to invest in sustainable energy when regulations are unclear, inconsistent, or overly complex [34]. The regulatory requirements and policies are still in the premature stages in developing countries like, China. In developing nations, there are no clear policy statements for sustainable energy plans which aligns with political visions [80].

The study also found that ecological conditions and lack of clear communication discourages sustainable energy technologies adoption. Some places may have restricted access to sustainable energy resources such as water, wind, or sunshine that impact the effectiveness of green technologies [55]. For instance, solar power is less effective in areas that observe regular cloud cover [97,98]. The adoption of sustainable energy technologies is largely dependent on effective communication as well [99]. The complexity of these technologies as well as misconceptions about costs and benefits can cause confusion and resistance among key stakeholders [34,74]. For instance, consumers, businesses, and policymakers all require different types of information to make informed decisions about new technologies. However, when the information is unclear or strongly technical then it fails to motivate all stakeholders towards green energy technologies [85].

At level three, the study identified that lack of energy storage technologies and lack of consumer paying capacity are the barriers of sustainable energy implementation. In line with previous studies, our study investigate that one of the major barrier of sustainable energy technologies is a shortage of advanced energy storage systems [100]. Since sustainable energy sources like sun and wind rarely provide electricity therefore, storage solutions are necessary to guarantee a steady and reliable power supply. When consumers perceive green technologies may not bring regular benefits then their paying capacity for green products and services is diminishing [11,101].

Next level two, the study found that barriers such as, lack of skilled personnel, insufficient knowledge, lack of research facilities, technology complexity and lack of trust and reliability on energy sources are deterring sustainable energy technologies [102]. The green energy transition would be slow if the workforce is not well trained [86,103]. The adoption of sustainable energy technology can be severely hindered by a lack of research and educational resources [104]. First and foremost, education is essential for acknowledging the advantages and possibilities of sustainable energy resources. People may be reluctant to embrace new technologies because they do not completely comprehend the importance of switching to sustainable energy sources [105]. Furthermore, a lack of research facilities might impede the development of innovative solutions. The lack of the focused and specialized research make it difficult for nations or areas to adopt effective policies for their green environment [14]. Many advanced technologies such as energy storage devices, wind turbines, and enhanced solar panels involves complex systems that require specific expertise to develop, install, and maintain [106]. Because of the complex nature, it may be challenging for people and institutions to accept sustainable energy technologies. Some companies can be severely hampered by a lack of confidence and dependability in energy sources and associated technologies [87]. Businesses are often hesitant to invest in sustainable energy due to concerns like technology’ long-term stability, cost, and dependability [107,108].

**Fig 3:**
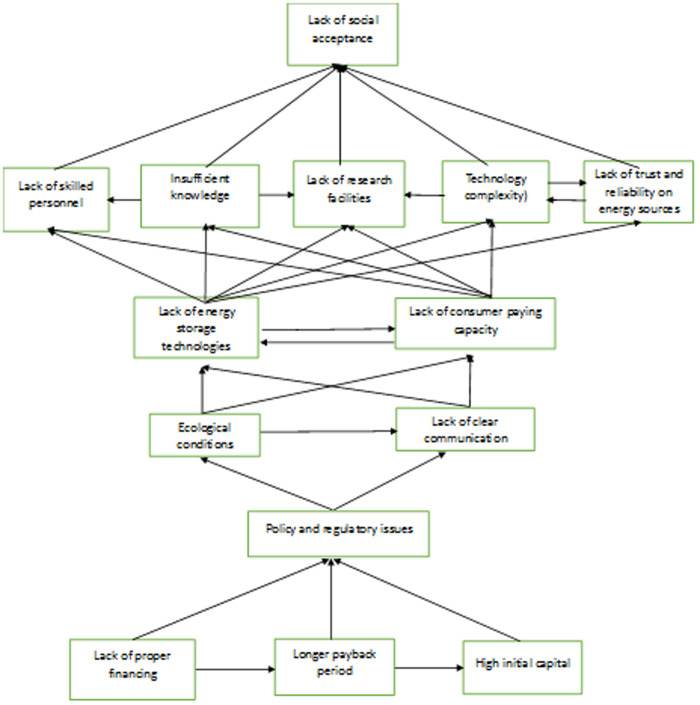
ISM model shows barriers of sustainable energy technologies adoption in hierarchy form.

Finally, the study found that lack of social acceptance located at level sixth is less critical barrier which discourages sustainable energy technologies adoption [109]. Lack of societal acceptance can hinder advancement of sustainable energy technology, as public backlash negatively affect firms’ willingness to adopt them [110]. The fear of environmental effects or the social stigma associated with new technology might also impact widespread acceptance [111]. The hierarchy-based structure of the mentioned barriers is shown in Fig 3.

### Study implication

The study offers several implications. First, the presentation of barriers to sustainable energy technologies through ISM framework shows the severity of barriers which can easily identify main barriers. Like, high initial capital cost, longer payback period, and lack of proper financing are the key barriers in the adoption of sustainable energy technologies so, these aforementioned problems can be resolved through devising effective policies. The tax credits, grants, or subsidies can lower the upfront costs for businesses [112,113]. The financial incentives enable the firms transition to renewable energy more accessible which can ease the economic burden on early adopters [114]. Local government can also enact policies to encourage long-term investment in sustainable energy, like green bonds, low-interest loans, and public-private partnerships [90]. With easily accessible financing options, the high costs and investments issues can be managed over time [39]. Social acceptability also affects public attitudes and support for legislative initiatives which are critical for the broad adoption of sustainable energy solutions. Communities are more inclined to support and embrace technology when they perceive its benefits. Even the most economical and successful technologies may face resistance if they are not socially acceptable so, social acceptance can play active role in Chinese green technological system. Stakeholders should concentrate on foundational interventions to shift linkage barriers toward a more manageable (dependent) position. These include funding workforce training and public awareness initiatives to lessen the shortage of skilled workers and inadequate knowledge, as well as encouraging research and development or subsidies to address the complexity of technology and energy storage constraints. Reducing the inability to pay and the lack of trust in energy sources may be achieved by increasing customer trust and financial assistance systems. This will stabilize these obstacles and lessen their negative effects.

Clear policy instruments and incentives can support clean energy technologies in China because strict rules and low financial assistance are deterring sustainable energy technologies adoption. Chinese government may reduce the cost of sustainable energy technologies by investing in research and development (R&D) [115]. The energy storage efficiency and cost-effectiveness of green technologies can be enhanced through innovation driven by public private partnership [116]. Finally, the lack of knowledge must be addressed through education and training initiatives [117].

## Conclusions, limitations and future work

Developing nations must embrace sustainable energy technology on urgent basis. Because rapid industrialization and economic expansion in these countries leads to higher energy consumption and environmental deterioration. China is one of largest energy consumer in the region which desperately needs sustainable energy solutions. Although, China has taken sincere efforts to implement sustainable energy technologies but their implementation is marred with significant challenges. Therefore, this study presented a unique multi-criteria decision-making method for exploring the barriers to sustainable energy technologies adoption in Chinese manufacturing context. The study is novel as it applies Interpretive Structural Modeling (ISM) to identify and prioritize 14 barriers based on their relative importance and structural interdependencies—offering a more strategic understanding for policy and decision-making. The MICMAC analysis classified these barriers into four categories—linkage, autonomous, dependent, and independent—based on their driving power and dependence power. The study results found that high initial capital, longer payback period, and lack of proper financing are main barriers of sustainable energy technologies adoption which supports previous investigations. More, the study also identified that poor communication and geographical factors such as sunlight for solar energy or consistent wind patterns for wind energy restrain the adoption of sustainable energy technologies. The Chinese firms may not able to achieve sustainable energy without addressing key challenges like inadequate energy storage technologies, limited consumer paying capacity, shortage of skilled labor, insufficient education and research facilities, complex nature of technologies, lack of trust and reliability on energy sources and low social acceptance.

This study has few limitations which needs to be addressed. The expert opinions that form the basis of the study’s ISM model may be biased as a result of their assessments. However, the proposed ISM-based model needs statistical validations. The study findings have not been validated with other methodological approaches. To improve the reliability of expert opinions, future work should acknowledge the importance of more formal validation methods like, fuzzy logic or Delphi. Also, the root causes of the problem have not been examined with tools like ISHIKAWA -diagram using 5 whys which is capable for identification of cause-effect relationship among elements. Therefore, future studies should apply ISHIKAWA -diagram using 5 whys on diverse industries and segment them. While creating the ISM Model through literature research, the current study only proposed 14 barriers of sustainable energy technologies however, this may not be a comprehensive list of factors influencing their adoption. The study’s focus on the Chinese manufacturing sector might limits the generalizability of other industries. Thus, future research should examine a comprehensive list of parameters across various sectors such as transportation, construction, and services. A multi country comparison can improve the research by showing how barriers’ effect and structure varies in different political, economic, and technical situations. By identifying important elements, this comparative understanding enables more generalizable conclusions and assists in customizing policy measures to meet the requirements of individual countries.

## Supporting information

S1 File Questionnaire for collection of responses from experts’ group (DOCX)
