# Supplementary material for: Constraints to sustainable energy technology implementation: Insights from an emerging economy using ISM-MICMAC analysis
Source: PLoS One. 2025 Sep 3;20(9):e0331334. doi: 10.1371/journal.pone.0331334 (PMC12407484; doi:10.1371/journal.pone.0331334)
Supplement: S1 File — (DOCX) [file pone.0331334.s001.docx]

Table A1. Questionnaire for collection of responses from experts’ group

| **Sr. #** | **Variable *j***  **Variable *i*** | B1 | B2 | B3 | B4 | B5 | B6 | B7 | B8 | B9 | B10 | B11 | | B12 | | B13 | | B14 | |
| --- | --- | --- | --- | --- | --- | --- | --- | --- | --- | --- | --- | --- | --- | --- | --- | --- | --- | --- | --- |
| **B1** | High initial capital |  |  |  |  |  |  |  |  |  |  |  | |  | |  | |  | |
| **B2** | Longer payback period |  |  |  |  |  |  |  |  |  |  |  | |  | |  | |  | |
| **B3** | Ecological conditions |  |  |  |  |  |  |  |  |  |  |  |  | |  | |  | |  |
| **B4** | Lack of proper financing |  |  |  |  |  |  |  |  |  |  |  |  | |  | |  | |  |
| **B5** | Lack of clear communication |  |  |  |  |  |  |  |  |  |  |  |  | |  | |  | |  |
| **B6** | Lack of skilled personnel |  |  |  |  |  |  |  |  |  |  |  |  | |  | |  | |  |
| **B7** | Insufficient knowledge |  |  |  |  |  |  |  |  |  |  |  |  | |  | |  | |  |
| **B8** | Lack of social acceptance |  |  |  |  |  |  |  |  |  |  |  |  | |  | |  | |  |
| **B9** | Policy and regulatory issues |  |  |  |  |  |  |  |  |  |  |  |  | |  | |  | |  |
| **B10** | Lack of energy storage technologies |  |  |  |  |  |  |  |  |  |  |  |  | |  | |  | |  |
| **B11** | Lack of research facilities |  |  |  |  |  |  |  |  |  |  |  |  | |  | |  | |  |
| **B12** | Lack of consumer paying capacity |  |  |  |  |  |  |  |  |  |  |  |  | |  | |  | |  |
| **B13** | Technology complexity |  |  |  |  |  |  |  |  |  |  |  |  | |  | |  | |  |
| **B14** | Lack of trust and reliability on energy sources and technology |  |  |  |  |  |  |  |  |  |  |  |  | |  | |  | |  |
